# Butyrate-Producing Bacteria in Regulating Intestinal Motility: Potential and Challenges

**DOI:** 10.3390/microorganisms14102283

**Published:** 2026-10-08

**Authors:** Qihong Liu, Jiabao Song, Zhihong Huang, Lunan Hu, Yuqun Lin, Yaying Huang, Yancheng Xu, Xiao Ke, Xiaofeng Huang, Yanyu Lin

**Affiliations:** 1 The Second People’s Hospital Affiliated to Fujian University of Traditional Chinese Medicine, Fuzhou 350003, China; drlqh@fjtcm.edu.cn (Q.L.); fjtcmhxf@163.com (X.H.); 2 College of Integrated Chinese and Western Medicine, Fujian University of Traditional Chinese Medicine, Fuzhou 350122, China; 3 The People’s Hospital Affiliated of Fujian University of Traditional Chinese Medicine, Fuzhou 350009, China

**Keywords:** butyrate-producing bacteria, intestinal motility, butyrate, intestinal health

## Abstract

Intestinal motility serves as a core component of digestive system function, and its rhythmic contractions and relaxations directly influence food digestion, nutrient absorption, and fecal excretion. Imbalanced intestinal motility readily precipitates various gastrointestinal disorders, including chronic constipation and irritable bowel syndrome, severely compromising quality of life. In recent years, investigations in gut microbiota have reported that butyrate-producing bacteria constitute a functional bacterial group capable of metabolizing dietary indigestible carbohydrates to synthesize butyrate. Through their metabolic product butyrate and multi-dimensional regulatory pathways, these bacteria play an indispensable role in maintaining intestinal motility homeostasis. This review provides an overview of the biological classification of butyrate-producing bacteria, mechanisms of butyrate production in the digestive tract, regulatory effects on intestinal motility, and recent application progress, as well as the current research status and intervention strategies in related diseases, aiming to elucidate the interaction mechanisms between butyrate-producing bacteria and intestinal motility, and to provide a theoretical basis for the prevention and treatment of intestinal motility disorders.

## 1. Introduction

### 1.1. Overview of Butyrate-Producing Bacteria

Butyrate-producing bacteria are a class of intestinal bacteria that produce butyrate as their primary metabolic end product. They convert indigestible carbohydrates from food into butyrate and maintain intestinal homeostasis through multiple biological functions, including providing energy to intestinal epithelial cells, influencing the intestinal mucosal barrier, regulating the gut microbiota, and modulating host immunity [1]. They are widely distributed throughout the digestive tract, including the rumen, oral cavity, colon, and cecum. As early as 1930, McCoy et al. discovered that butyrate-producing bacteria can generate butyrate through various fermentation pathways (fermentation of carbohydrates, polyols, and glycosides; fermentation of lactate; and fermentation of proteins and their derivatives), a process accompanied by the production of other metabolites such as acetate, alcohols, and acetone [2]. The metabolic products of butyrate-producing bacteria include short-chain fatty acids (SCFAs: acetate, propionate, and butyrate) [2,3], enzymes (proteases, amylases, lipases, glycosidases, and cellulases) [2,4], and B vitamins, among which butyrate is the principal metabolic product of butyrate-producing bacteria and serves as the primary mediator of their functions, playing an important role in promoting intestinal development and maintaining intestinal homeostasis [5].

### 1.2. Representative Butyrate-Producing Bacteria

Butyrate-producing bacteria represent a functional group within the host’s gut microbiota rather than a monophyletic phylogenetic cluster. Their diversity and functionality are important manifestations of the role of gut microbiota. Vital et al. [6] screened 3184 sequenced bacterial genomes from an integrated microbial genome database and identified 225 bacterial genomes potentially capable of producing butyrate, including many previously unknown candidate genes. Various types of butyrate-producing bacteria exist in the intestine, predominantly anaerobic, Gram-positive bacteria, mostly belonging to the phylum *Firmicutes*, specifically the families *Clostridiaceae*, *Lachnospiraceae*, and *Oscillospiraceae* (formerly included within *Ruminococcaceae*), which are widely present in the cecum and colon of the animal digestive tract [7,8].

Bacteria of the family *Clostridiaceae* are found in diverse environments, including soil, water bodies, the intestinal tracts of animals and plants, and the decomposition processes of other organic wastes. As model microorganisms, they facilitate in-depth understanding of the ecology, metabolic pathways, and gene regulation of anaerobic bacteria. The genus *Clostridium* serves as the type genus of this family. Also known as the spindle-shaped spore-forming genus, *Clostridium* belongs to the phylum Bacillota (formerly Firmicutes) and family *Clostridiaceae*; most species are saprophytic. Its representative species, *Clostridium butyricum* (also known as butyric acid clostridium or butyrate-producing clostridium), is a typical intestinal commensal bacterium and a strictly anaerobic Gram-positive bacterium that may appear Gram-negative in later culture stages. Its metabolic products include butyrate, acetate, propanediol, and hydrogen. Renowned for its potent butyrate-producing capacity, it is also the typical model organism of the genus *Clostridium* and has been used to intervene in and prevent human diarrhea and gut microbiota disturbances, as well as to enhance humoral immune responses [9]. The family *Lachnospiraceae* comprises important anaerobic bacteria in the intestine, exhibiting various biological characteristics such as producing SCFAs, participating in bile acid metabolism, and resisting pathogen invasion, thereby exerting certain effects on host health. The *Lachnospiraceae* family includes multiple genera capable of butyrate synthesis, such as *Roseburia* and *Coprococcus*. *Roseburia* can improve intestinal biodiversity, enhance glucose tolerance, aid in weight reduction, revitalize colonic cells, and may serve as a biomarker for symptomatic pathologies (such as gallstone formation) or as a probiotic to restore beneficial flora [10]. *Coprococcus* possesses the ability to ferment carbohydrates and generate butyrate and acetate. It can be used as a microbial marker to assess gastrointestinal health in humans, is negatively correlated with colitis and inflammatory colorectal cancer, and may have potential anti-inflammatory and anti-tumor effects [11].

The family *Oscillospiraceae* (formerly classified within *Ruminococcaceae*) is primarily found in the rumen of ruminant animals but has also been discovered in the intestines of other mammals. Bacteria of this family play important roles in the digestive systems of animals, particularly in the degradation of cellulose and other complex polysaccharides. *Ruminococcus* is a Gram-positive, anaerobic, spherical, non-spore-forming bacterium and was one of the earliest bacteria discovered in the rumen, playing a crucial role in metabolism. *Ruminococcus* obtains nutrients by decomposing cellulose in the host’s digestive system and can also ferment glucose and xylose, digesting resistant starch. Its metabolic product butyrate can repair colonic mucosal damage and exerts certain auxiliary intervention effects on intestinal diseases (IBS, IBD, etc.) [12]. *Faecalibacterium* was one of the first commensal bacterial genera found to differ between individuals with and without inflammatory bowel disease (IBD), and its beneficial effects appear to be related to its anti-inflammatory properties [13].

### 1.3. Mechanisms of Butyrate Production

**Figure 1:**
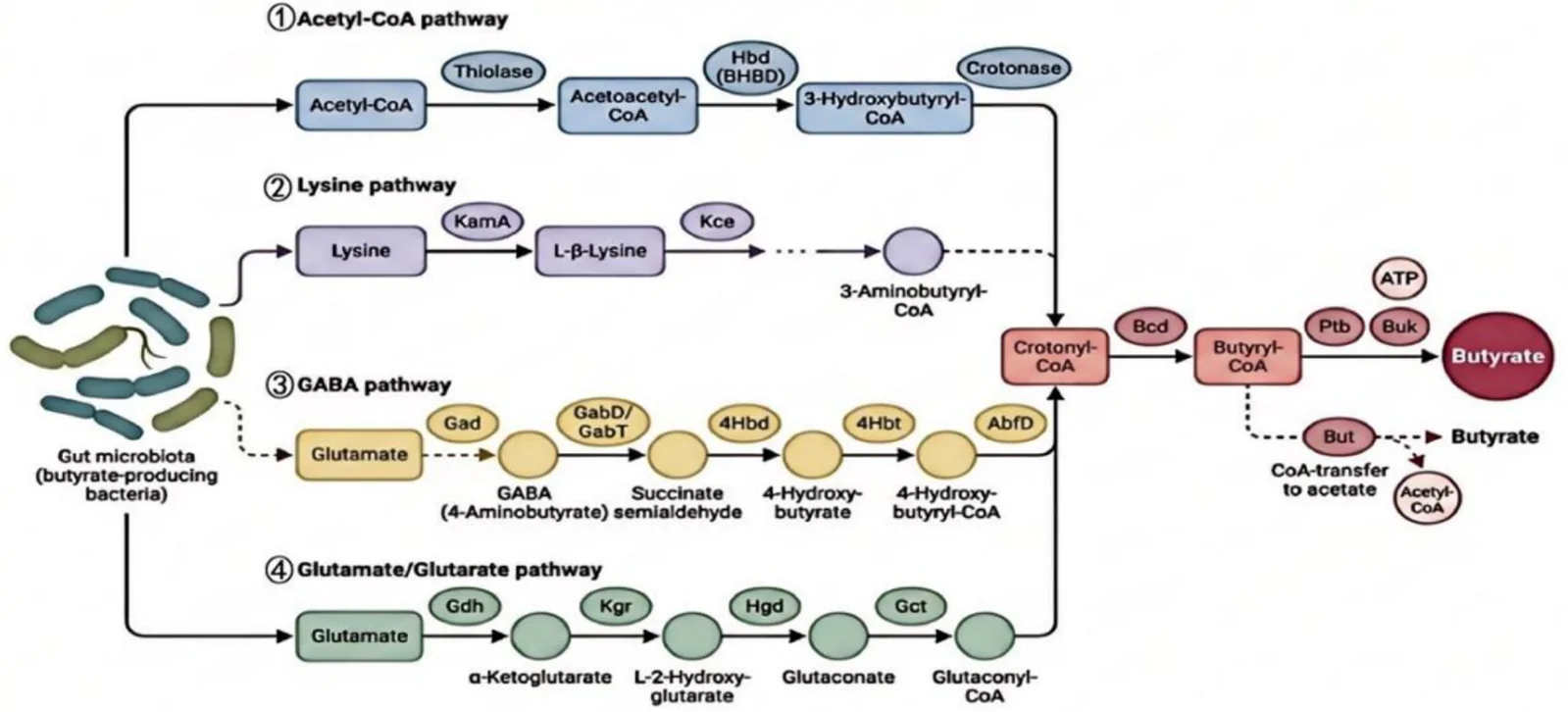
Butyrate-producing mechanism of butyrate-producing bacteria. ① Acetyl-CoA pathway: Acetyl-CoA stands for acetyl-coenzyme A; Thiolase stands for acetyl-CoA acetyltransferase; Hbd/BHBD stands for 3-hydroxybutyryl-CoA dehydrogenase (the same enzyme listed under alternative names); Crotonase stands for enoyl-CoA hydratase. ② Lysine pathway: KamA stands for lysine-2,3-aminomutase; Kce stands for 3-keto-5-aminohexanoate cleavage enzyme. ③ 4-Aminobutyrate (GABA) pathway: GAD stands for glutamate decarboxylase; GABA stands for γ-aminobutyric acid; GabD stands for succinate semialdehyde dehydrogenase; GabT stands for succinate semialdehyde aminotransferase; AbfH/4Hbd stands for 4-hydroxybutyrate dehydrogenase (the text uses AbfH, while the figure uses 4Hbd); 4Hbt stands for 4-hydroxybutyryl-CoA transferase; AbfD stands for 4-hydroxybutyryl-CoA dehydratase. ④ Glutamate/Glutarate pathway: Gdh stands for glutamate dehydrogenase; Kgr stands for α-ketoglutarate reductase; Hgd stands for 2-hydroxyglutarate dehydrogenase; Gct stands for glutaconate CoA-transferase. Common downstream enzymes: Bcd stands for butyryl-CoA dehydrogenase; Ptb stands for phosphotransbutyrylase; Buk stands for butyrate kinase; But stands for butyryl-CoA:acetate CoA transferase; ATP stands for adenosine triphosphate; CoA stands for coenzyme A.

One of the key pathways through which butyrate-producing bacteria exert their effects is by transporting the active molecule (butyrate) to specific intestinal sites to exert targeted regulatory actions; therefore, the mechanisms underlying butyrate biosynthesis have attracted substantial research attention. In recent years, researchers have summarized four butyrate-producing pathways: the acetyl-CoA pathway, the lysine pathway, the glutarate pathway, and the 4-aminobutyrate pathway. Among these, the acetyl-CoA pathway is the most common, followed by the lysine pathway [6], as specifically illustrated in Figure 1.

Carbohydrates ingested by the human body undergo digestion and absorption in the small intestine before reaching the colon. These complex polysaccharides (such as starch and xylan) are utilized by butyrate-producing bacteria through substrate-level phosphorylation to generate ATP. During this process, carbohydrates produce intermediate metabolites such as pyruvate and acetyl-CoA. Two molecules of acetyl-CoA are condensed by thiolase to form acetoacetyl-CoA. Thiolase is encoded by two gene variants, type A (KAT type) and type B (ACAT type). Type A refers to class I thiolase (3-ketoacyl-CoA thiolase, KAT), whereas type B refers to class II thiolase (acetoacetyl-CoA thiolase, ACAT). Subsequently, under the catalysis of 3-hydroxybutyryl-CoA dehydrogenase (BHBD), 3-hydroxybutyryl-CoA is generated. 3-hydroxybutyryl-CoA is converted to crotonyl-CoA by crotonase catalysis. The step from crotonyl-CoA to butyryl-CoA represents the central pathway of the entire route, as this step is also the common pathway shared by the other three pathways [14]. In this step, the electron transfer flavoprotein complex of butyryl-CoA dehydrogenase provides electrons, thereby helping butyrate-producing bacteria conserve energy; this reaction may also be closely related to fatty acid oxidation in prokaryotes and eukaryotes [15]. The final step from butyryl-CoA to butyrate is catalysis by butyryl-CoA:acetate CoA-transferase (encoded by the *But* gene) or butyrate kinase (encoded by the *Buk* gene, following phosphorylation of butyryl-CoA). Typically, these two genes are used as biomarkers for identifying/detecting butyrate-producing bacterial communities [16]. Currently, real-time PCR detection methods for these genes can be established using degenerate primers, thereby enabling semi-quantitative monitoring of dynamic changes in butyrate-producing bacteria present in the human colon microbiota.

In addition to the acetyl-CoA pathway, the other three butyrate-producing pathways all utilize amino acids as primary substrates, with the lysine pathway being the most representative [6]. Bui et al. [17] utilized ^13^C-NMR isotope tracing and discovered that butyrate-producing bacteria can cleave the C2 and C3 residues of lysine. NMR results showed that lysine is almost completely converted to an equimolar amount of butyrate, and intermediate products of the acetyl-CoA pathway are generated during this process. From gene expression analysis, genes related to the lysine pathway include one operon-like cluster (containing genes for extracting and utilizing fructoselysine and psicoselysine) [18] and one lysine utilization operon (encoding six enzymes involved in the conversion of lysine to butyrate). Furthermore, through genomic analysis of *Enterobacter* AF211, all 14 enzymes related to the lysine pathway were predicted, including CoA transferase *AtoD-A*, 4Hbt, and But. Further activity induction and proteomic analysis revealed that *AtoD-A* is highly expressed under lysine degradation conditions. *AtoD-A* is also considered the key enzyme of the lysine pathway. As this represents the first description of the lysine pathway in the intestinal ecosystem, the lysine utilization operon and the identified *AtoD-A* gene may serve as markers for the occurrence of the lysine pathway.

Apart from the central pathway (crotonyl-CoA to butyryl-CoA), the glutarate pathway and the 4-aminobutyrate pathway share no common substrates with other pathways, and these two pathways account for only a small proportion of total butyrate production (ranging from 2.4 to 2.5%) through a metagenomics-based assessment [6], indicating that these two butyrate-producing pathways are relatively independent and their intermediate metabolites are not preferentially utilized by most butyrate-producing bacteria. 4-Aminobutyrate is converted to vinyl-acetyl-CoA under the actions of 4-hydroxybutyrate dehydrogenase (AbfH), 4-hydroxybutyrate CoA transferase (4Hbt), and 4-hydroxybutyryl dehydratase (AbfD). The glutarate pathway involves the participation of glutamate dehydrogenase, 2-hydroxyglutarate dehydrogenase (L2Hgdh), glutaconate-CoA transferase (Gct), 2-hydroxyglutaryl-CoA dehydrogenase (HgCoAd), and glutaconyl-CoA decarboxylase (Gcd) [19]. Both pathways similarly converge at the central pathway. Notably, glutarate can be converted to 4-aminobutyrate through glutamate decarboxylase under the utilization by anaerobic bacteria [20].

## 2. Regulatory Roles of Butyrate-Producing Bacteria in Intestinal Motility Disorders

Intestinal motility disorders are classified as primary or secondary, with the former including irritable bowel syndrome (IBS), functional constipation, and chronic intestinal pseudo-obstruction. With changing lifestyles, the incidence of intestinal motility disorders has been increasing year by year, imposing a growing socioeconomic and medical burden [21]. Intestinal microecological disturbances participate in multiple pathological processes; clinically, interventions often include dietary modifications, probiotics and prebiotics, and fecal microbiota transplantation (FMT). Studies have found that the metabolites of butyrate-producing bacteria play important roles in colonic motility, although controversy remains. Butyrate-producing bacteria exert biological effects through the metabolite butyrate, which serves as a core signaling molecule regulating intestinal peristalsis, colonic transit, and smooth muscle rhythm. However, the regulatory effects of different concentrations of butyrate on colonic motility are complex. The effects of butyrate on colonic motility are concentration-dependent: in ex vivo rat colon, concentrations ≤ 10 mM tend to increase contractile activity, whereas concentrations ≥ 30 mM inhibit contractile activity and reduce mucin/water absorption. The honest version of the argument is that the inhibitory effect is demonstrated ex vivo at concentrations that may be reached locally—near the mucosal surface, in the proximal colon after a large fermentable load, or with a delayed-release formulation delivering a bolus—but that whether it occurs in the intact human colon under ordinary conditions is unknown [22,23,24].

**Figure 2:**
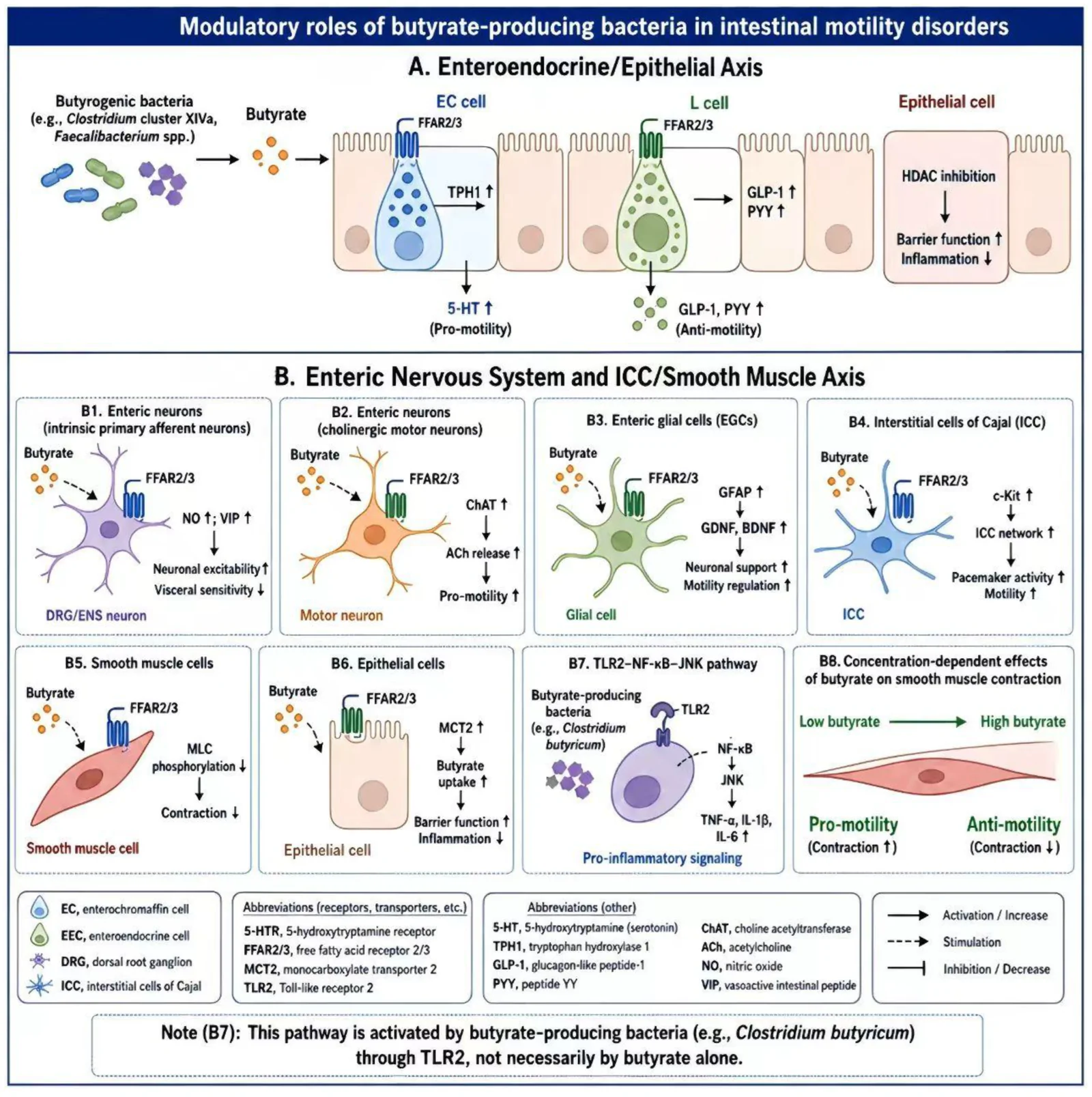
Modulatory roles of butyrate-producing bacteria in intestinal motility disorders. (**A**) Enteroendocrine/epithelial axis: butyrate released by butyrate-producing bacteria activates enterochromaffin (EC) cells and L cells via FFAR2/FFAR3, regulating the release of 5-HT, GLP-1, and PYY. (**B**) Enteric nervous system and ICC/smooth muscle axis: butyrate acts on enteric neurons, enteric glial cells (EGCs), interstitial cells of Cajal (ICCs), and smooth muscle cells through multiple receptors and signaling pathways to modulate intestinal motility. The TLR2–NF-κB–JNK pathway (B7) is activated by butyrate-producing bacteria such as Clostridium butyricum, not necessarily by butyrate alone. (G) Concentration-dependent bidirectional effect of butyrate on smooth muscle contraction: low butyrate enhances contraction (pro-motility), whereas high butyrate inhibits contraction (anti-motility). EC, enterochromaffin cell; EEC, enteroendocrine cell; 5-HT, 5-hydroxytryptamine; 5-HTR, 5-hydroxytryptamine receptor; TPH1, tryptophan hydroxylase 1; GLP-1, glucagon-like peptide-1; PYY, peptide YY; ENS, enteric nervous system; ICCs, interstitial cells of Cajal; ChAT, choline acetyltransferase; ACh, acetylcholine; MCT2, monocarboxylate transporter 2; TLR, Toll-like receptor; NF-κB, nuclear factor kappa-light-chain-enhancer of activated B cells; JNK, c-Jun N-terminal kinase; TNF-α, tumor necrosis factor-α; IL, interleukin; GFAP, glial fibrillary acidic protein; GDNF, glial cell line-derived neurotrophic factor; BDNF, brain-derived neurotrophic factor; NO, nitric oxide; VIP, vasoactive intestinal peptide; DRG, dorsal root ganglion; FFAR2/3, free fatty acid receptor 2/3; HDAC, histone deacetylase; MLC, myosin light chain.

Therefore, it is necessary to further explore the role of butyrate in the intestine, as its effects on intestinal motility are likely mediated through multiple pathways and targets. See Figure 2 for details.

### 2.1. Acting on Enteroendocrine Cells to Regulate Intestinal Hormone Secretion

Enteroendocrine cells (EECs) are scattered throughout the intestinal epithelium and constitute the largest endocrine organ of the intestine, accounting for approximately 1% of intestinal epithelial cells [25,26]. The metabolite butyrate from butyrate-producing bacteria can activate free fatty acid receptors (FFARs), particularly FFAR2/GPR43 and FFAR3/GPR41, on the surface of EECs, thereby regulating the synthesis and release of various gastrointestinal hormones and subsequently influencing intestinal motility [24].

#### 2.1.1. Enterochromaffin Cells (ECs): Releasing 5-HT to Promote Intestinal Propulsive Motility

Enterochromaffin cells (ECs) are the primary source of 5-hydroxytryptamine (5-HT) in the intestine, accounting for over 90% of intestinal 5-HT synthesis. ECs can recognize short-chain fatty acids (including acetate, propionate, and butyrate) produced by the gut microbiota and upregulate the expression of tryptophan hydroxylase 1 (TPH1) through signaling pathways mediated by FFAR2/GPR43 and FFAR3/GPR41 receptors, thereby promoting 5-HT synthesis and release [27,28]. Released 5-HT can activate 5-HT_4_ receptors expressed on intestinal neurons and smooth muscle cells, enhance cholinergic neurotransmission, and promote gastrointestinal peristaltic reflexes and intestinal propulsive motility [29]. Studies have shown that reduced abundance of butyrate-producing bacteria in the intestine of constipation patients is accompanied by decreased fecal butyrate levels, while supplementation with butyrate or butyrate-producing bacteria can significantly increase colonic 5-HT content and accelerate colonic transit [24,30,31]. Furthermore, as a core signaling molecule in the brain–gut axis, changes in 5-HT secretion levels may also influence central regulation of intestinal motility through vagal afferent fibers. Enterochromaffin cells can recognize short-chain fatty acids produced by the gut microbiota and generate 5-HT, thereby promoting gastrointestinal motility through activation of 5-HT_4_ receptors expressed on intestinal neurons.

#### 2.1.2. L Cells: Secreting GLP-1 and PYY Leads to Negative Feedback and Inhibits Peristalsis

L cells are mainly distributed in the ileum and colon and are the primary secretory cells of glucagon-like peptide-1 (GLP-1) and peptide YY (PYY) [32]. Under pathological conditions, GLP-1 overexpression is associated with significantly prolonged gastrointestinal transit. Studies have reported that germ-free mice or mice treated with antibiotics show reduced intestinal butyrate concentrations, increased expression of proglucagon (Gcg) in colonic L cells, and elevated plasma GLP-1 levels [33]. Additionally, PYY, another satiety hormone secreted by L cells, can inhibit intestinal peristalsis and gastric acid secretion and prolong gastric emptying time by activating Y2 receptors [34]. The regulation of L cell hormone secretion by butyrate exhibits complex concentration-dependent and site-specific characteristics. Low concentrations of butyrate may promote PYY release through FFAR2 activation, whereas high concentrations of butyrate may inhibit excessively secreted gastrointestinal hormones through other mechanisms to maintain intestinal motility balance [35].

### 2.2. Regulating the Enteric Nervous System to Reshape Intestinal Neural Excitation Homeostasis

The enteric nervous system (ENS) is known as the “second brain” of the human body, composed of approximately 100 million neurons that can independently regulate intestinal motility, secretion, and blood flow locally without relying on the central nervous system [36]. The metabolite butyrate from butyrate-producing bacteria can regulate the structure and function of the ENS through multiple pathways, thereby reshaping intestinal neural excitation homeostasis.

#### 2.2.1. Promoting ENS Development and Neuronal Phenotype Differentiation

Studies have shown that the gut microbiota and its metabolites are crucial for ENS development and maturation. Germ-free (GF) neonatal mouse models have demonstrated that the absence of gut microbiota can lead to reduced ENS neuron numbers and abnormal proportions of neural subtypes, thereby affecting intestinal motility function [37]. As a key microbial metabolite, butyrate can promote ENS development through dual mechanisms involving energy substrate provision and epigenetic regulation. On one hand, butyrate can be taken up by ENS neurons and enhance the excitability of choline acetyltransferase (ChAT)-positive excitatory neurons, promoting acetylcholine release to accelerate colonic transit [38]. On the other hand, butyrate acts as a histone deacetylase (HDAC) inhibitor, regulating the expression of neurodevelopment-related genes and promoting neuronal differentiation and synaptic plasticity [39]. Caetano et al. reported that GPR41 is expressed in intestinal interneurons, and butyrate can bind to GPR41 to alleviate experimentally induced ulcerative colitis-related neuronal loss and glial cell proliferation, improving the morphology and functional integrity of the intestinal neural network [40].

#### 2.2.2. Regulating Enteric Glial Cell Function and Neuroinflammation

Enteric glial cells (EGCs) are the most abundant cell type in the ENS and play key roles in maintaining intestinal immune homeostasis, barrier integrity, and neuroprotection [41]. Butyrate can regulate the secretion of neurotrophic factors and inflammatory responses in EGCs by activating Toll-like receptors (TLRs) and GPR receptors on their surfaces. In intestinal motility disorders, abnormal activation and reactive gliosis of EGCs can lead to neuroinflammation and neuronal damage [42]. Butyrate reduces the expression of pro-inflammatory factors such as tumor necrosis factor-α (TNF-α) and interleukin-6 (IL-6) by inhibiting HDAC activity, thereby alleviating damage to the ENS from neuroinflammation [43]. Additionally, butyrate can activate intrinsic and extrinsic afferent neurons in the dorsal root ganglia, enhancing intestinal sensory afferent signals and regulating brain–gut axis neural conduction [44].

#### 2.2.3. Regulating Neurotransmitter Balance

The ENS regulates intestinal smooth muscle contraction through the release of various neurotransmitters. The balance between excitatory neurotransmitters (such as acetylcholine and substance P) and inhibitory neurotransmitters (such as nitric oxide and vasoactive intestinal peptide) determines the rhythm and intensity of intestinal movement [45]. Butyrate can regulate intestinal neural excitability by influencing the synthesis, release, and receptor expression of neurotransmitters. Meanwhile, butyrate can also maintain the fine balance of intestinal motility by regulating serotonergic, cholinergic, and nitrergic neurotransmission [45]. Studies have shown that Clostridium butyricum, as a butyrate-producing probiotic, can promote intestinal motility by modulating enteric neurotransmission and interstitial cells of Cajal [46].

### 2.3. Regulating ICCs and Intestinal Smooth Muscle to Maintain Rhythmic Contraction

The rhythmic contraction of intestinal smooth muscle depends on the coordinated interaction among the enteric nervous system, interstitial cells of Cajal (ICCs), and smooth muscle cells [47]. As the pacemaker cells of the intestine, ICCs generate and propagate slow-wave potentials, regulating the frequency and rhythm of smooth muscle contraction; simultaneously, ICCs serve as intermediaries in neuromuscular signal transmission, amplifying and integrating excitatory and inhibitory signals from the ENS [48]. The metabolite butyrate from butyrate-producing bacteria can affect ICCs and smooth muscle function through direct or indirect pathways.

#### 2.3.1. Protecting ICC Network Structure and Function

The integrity of the ICC network is fundamental to maintaining normal intestinal motility. In intestinal motility disorders such as slow-transit constipation and diabetic gastroparesis, reduced ICC numbers and disrupted network structure are common pathological features [49,50]. First, butyrate upregulates c-Kit expression in ICCs through HDAC inhibition, supporting ICC survival and network integrity [51]. Second, butyrate influences ICC function through c-kit/SCF pathway activation, thereby facilitating synchronized contractile activity [52]. Furthermore, butyrate may maintain ICC network homeostasis by suppressing intestinal inflammatory responses [53]. *Clostridium butyricum*, as a butyrate-producing probiotic, can increase ICC viability and promote intestinal motility through TLR2-dependent activation of nuclear factor (NF)-κB and c-Jun N-terminal kinase (JNK) signaling pathways [46].

#### 2.3.2. Regulating Smooth Muscle Contractile Activity

Butyrate exerts direct regulatory effects on intestinal smooth muscle contraction. Studies have shown that butyrate can directly stimulate colonic smooth muscle contraction, an effect that persists in the presence of tetrodotoxin, suggesting a non-neuronal component [54]. In addition, SCFAs including butyrate can modulate smooth muscle contractility, although the precise receptor mechanisms on intestinal smooth muscle cells remain to be fully elucidated [55]. However, the effects of butyrate on smooth muscle exhibit marked concentration dependence: low concentrations of butyrate can enhance colonic motility and promote intestinal content propulsion, whereas high concentrations of butyrate may cause smooth muscle cell membrane hyperpolarization by activating potassium ion channels, inhibiting intestinal water and electrolyte absorption and mucin secretion, and weakening colonic motility [54]. This bidirectional regulatory effect suggests that the physiological role of butyrate in the intestine is closely related to its local concentration, intestinal region, and disease state.

#### 2.3.3. Integrating Multi-Cellular Regulatory Networks

Intestinal motility regulation involves a complex network comprising the ENS, ICC, smooth muscle cells, and immune cells [47]. As a key signaling molecule in this network, butyrate can achieve synergistic regulation by simultaneously acting on multiple targets. For example, butyrate promotes 5-HT release by activating ECs; 5-HT subsequently acts on ICC and smooth muscle cells. Simultaneously, butyrate enhances the excitability of ChAT-positive neurons in the ENS, promoting acetylcholine release, which acts on muscarinic receptors on ICC and M_3_ receptors on smooth muscle cells to coordinate intestinal contraction [56]. Additionally, butyrate can indirectly protect ICC and smooth muscle function by regulating the intestinal immune microenvironment, reducing mast cell degranulation and inflammatory mediator release [53]. This multi-pathway, multi-target integrated regulatory model fully demonstrates the central role of butyrate-producing bacteria in maintaining intestinal motility homeostasis.

## 3. Butyrate-Producing Bacteria in Specific Intestinal Motility Disorders

### 3.1. Irritable Bowel Syndrome (IBS)

The relationship between butyrate-producing bacteria and IBS is complex. A meta-analysis by Sun et al. of nine case–control studies (472 IBS patients and 228 healthy controls) found no significant difference in fecal butyrate levels between IBS patients and healthy controls (SMD = −0.21, 95% CI: −0.45 to 0.03), and no significant differences among IBS subtypes [52]. This null result highlights that fecal SCFA concentration is a poor proxy for colonic production and turnover, since it reflects the net of synthesis, colonocyte uptake, and transit. Nevertheless, subtype-stratified and taxon-level analyses suggest divergent patterns: butyrate-producing taxa such as the Roseburia–Eubacterium rectale complex are reduced in IBS-C [51], while IBS-D can be associated with relative elevations of fecal butyrate and increased sulfate-reducing bacteria [57]. IBS-C is characterized by delayed colonic transit, and lower fecal butyrate levels in IBS-C may attenuate butyrate-mediated stimulation of colonic motility [58]. In contrast, IBS-D is associated with accelerated transit and microbial dysbiosis, which may contribute to disturbed contractility [59,60].

Clinical studies on fecal microbiota transplantation (FMT) have further reported the central role of butyrate-producing bacteria. El-Salhy et al. conducted a randomized, double-blind, placebo-controlled trial enrolling 165 IBS patients, transplanting 30 g or 60 g of donor feces into the distal duodenum of patients via gastroscopy [61]. Results showed that at 3 months post-FMT, the response rate (IBS-SSS score reduction ≥ 50 points) was 76.9% in the 30 g group and 89.1% in the 60 g group, significantly higher than the autologous feces control group (23.6%) [61]. At 1 month post-FMT, patient fecal butyrate levels were significantly elevated (30 g group: 10.6 ± 8.0 → 15.5 ± 10.2 mmol/kg, *p* = 0.005; 60 g group: 12.4 ± 8.9 → 17.8 ± 14.3 mmol/kg, *p* = 0.01), with greater elevations observed in responders; butyrate levels were significantly negatively correlated with IBS-SSS score (*p* = 0.005, r = −0.4) and fatigue scores (*p* = 0.005, r = −0.3), suggesting that FMT improves motility disorders through reconstruction of gut microbiota, increased abundance of butyrate-producing bacteria, and subsequent butyrate-mediated neural regulation and barrier repair [62].

Notably, the imbalance of butyrate-producing bacteria in IBS patients is also closely related to psychological status. Liu et al. performed 16S rRNA sequencing and metabolomic analysis on 70 IBS-D patients and 46 healthy controls, finding that the abundance of butyrate-producing genera such as *Dialister* and *Roseburia* was significantly lower in IBS-D patients than in healthy controls, and *Dialister* abundance was significantly negatively correlated with anxiety scores (SAS, r = −0.347, *p* = 0.003), depression scores (SDS, r = −0.308, *p* = 0.010), and IBS symptom severity (r = −0.285, *p* = 0.017) [63]. This suggests that deficiency of butyrate-producing bacteria may exacerbate the bidirectional deterioration of emotional disorders and intestinal symptoms through the gut–brain axis, and clinical treatment requires comprehensive intervention combining psychological intervention and flora regulation.

### 3.2. Functional Constipation

Research by Parthasarathy et al. found that the abundance of butyrate-producing bacteria such as *Faecalibacterium prausnitzii* in the feces of functional constipation patients was significantly lower than in healthy individuals, and its abundance was negatively correlated with prolonged colonic transit time and constipation symptom severity [64]. Intervention strategies supplementing butyrate-producing bacteria or promoting their proliferation can significantly improve constipation symptoms, with mechanisms closely related to butyrate activation of the ENS, promotion of 5-HT secretion, and increased fecal water content [65].

Clinical observations have shown that consuming prebiotic foods rich in resistant starch to promote the proliferation of butyrate-producing bacteria is a safe and effective strategy for improving functional constipation. Luk-In et al. [66] conducted a randomized, double-blind, placebo-controlled trial enrolling 98 chronic constipation patients, in which subjects were supplemented with 9 g of resistant starch type 3 (RS-3) daily for 12 weeks. Results showed that *Bifidobacterium* and *Akkermansia* were significantly increased in the RS-3 group. Although these taxa are not direct butyrate producers, the acetate and lactate they generate can be cross-fed to butyrogenic Firmicutes such as *Roseburia* and *Faecalibacterium*, thereby indirectly promoting colonic butyrate production. Most subjects achieved bowel movement frequencies of 4–5 times per week, Bristol Stool Scale (BSS) scores trended toward the healthy type (BSS = 4), and Constipation Scoring System (CSS) scores were significantly reduced. Bush et al. [67] conducted a three-arm randomized, double-blind, placebo-controlled clinical trial showing that healthy adults consuming 3.5 g of resistant potato starch (Solnul™, MSP Starch Products Inc., Carberry, Canada) daily for 4 weeks significantly increased the relative abundance of *Bifidobacterium* (*p* = 0.038) and *Akkermansia* (*p* = 0.014) and reduced diarrhea and constipation-related bowel movements. Subsequent secondary analysis by Bush et al. also reported that resistant potato starch improved abnormal bowel symptom-related indicators [67].

## 4. Therapeutic Strategies for Intestinal Motility Disorders

Based on the close association between butyrate-producing bacteria and intestinal motility, an intervention system centered on “proliferating existing flora, supplementing exogenous strains, and optimizing the metabolic environment” has been established, demonstrating promising prospects in clinical application.

### 4.1. Prebiotic-Targeted Feeding

Consuming specific substrates for butyrate-producing bacteria (prebiotics) to promote the proliferation of endogenous butyrate-producing bacteria in the intestine is the safest intervention method. Prebiotics are defined as “substrates that are selectively utilized by host microorganisms and confer health benefits.” Insoluble dietary fibers (such as cellulose in whole grains), fructooligosaccharides (FOS), and inulin can be utilized by butyrate-producing bacteria such as *Roseburia* and *Faecalibacterium* to produce butyrate. A randomized, double-blind, placebo-controlled trial conducted by De Giani et al. [68] showed that synbiotic preparations containing fructooligosaccharides and inulin could optimize gut microbiota structure and increase the abundance of butyrate-producing bacteria, suggesting that they may exert intestinal regulatory effects by promoting endogenous butyrate production. A randomized controlled trial by Laffin et al. [69] also reported that daily supplementation with high-amylose maize resistant starch (HAM-RS2) significantly increased the proportion of *Faecalibacterium* in the feces of end-stage renal disease patients.

Partially hydrolyzed guar gum (PHGG), as a soluble dietary fiber, has substantial evidence for improving intestinal function. Yasukawa et al. conducted a randomized, double-blind, placebo-controlled trial on 44 healthy volunteers with diarrhea tendency, showing that daily supplementation with 5 g PHGG for 3 months significantly improved Bristol Stool Scale scores, increased the abundance of *Bifidobacterium*, *Ruminococcus*, and *Megasphaera*, and maintained organic acid-producing bacteria such as *Sellimonas* at higher levels [70]. Although *Bifidobacterium* is not a direct butyrate producer, the acetate and lactate it generates can be cross-fed to butyrogenic taxa such as *Roseburia* and *Faecalibacterium*, thereby indirectly supporting colonic butyrate production.

However, differences among IBS subtypes must be noted: IBS-C patients exhibit delayed intestinal transit, and moderately increasing fermentable dietary fibers such as resistant starch can provide substrates for butyrate-producing bacteria, promoting butyrate generation to stimulate colonic motility. In contrast, IBS-D patients should avoid large amounts of rapidly fermentable oligosaccharides to prevent exacerbation of intestinal osmotic load and diarrhea. Furthermore, traditional fermented foods (such as natto, tempeh, and yogurt) not only contain active probiotics, but their fermentation-derived metabolites (such as organic acids and peptides) can also provide cross-feeding substrates for butyrate-producing bacteria and can serve as daily dietary supplementation strategies.

### 4.2. Butyrate-Producing Bacterial Supplementation

Direct supplementation with live bacterial preparations can rapidly modulate the gut microbiota. Currently, the most extensively studied strains include the direct butyrate producers *Clostridium butyricum* and *Faecalibacterium prausnitzii*, as well as *Akkermansia muciniphila*, which does not produce butyrate itself but can indirectly support colonic butyrate levels through mucin degradation and acetate cross-feeding of butyrogenic Firmicutes. Bertuccioli et al. conducted a clinical evaluation enrolling 51 IBS patients. In the IBS-D subtype, patients received the butyrate-producing probiotic *Clostridium butyricum* CBM588; in the IBS-C subtype, patients received *Bifidobacterium longum* W11. After 3 months, IBS-SSS scores were significantly reduced in both groups, although the effect in the IBS-C arm may reflect mechanisms other than direct butyrogenesis [71]. Meynier’s research in an IBS mouse model also reported that pasteurized *A. muciniphila* significantly reduced colonic hypersensitivity, enhanced intestinal barrier function (upregulated ZO-1 expression), and improved anxiety-like behavior, suggesting its multi-target effects through the gut–brain axis [72].

However, oral live bacterial preparations face challenges such as destruction by gastric acid and bile and low intestinal colonization rates. Microencapsulation embedding technology is a key strategy to address these issues. Microencapsulated sodium butyrate, a formulation designed for colonic delivery, has also shown symptomatic benefit in IBS patients. In a randomized, double-blind, placebo-controlled trial, Banasiewicz et al. reported that 300 mg/day of microencapsulated sodium butyrate for 12 weeks significantly reduced the frequency of abdominal pain during defecation and improved defecation disorder and bowel habits compared with placebo, with no treatment-related adverse events reported among the 66 enrolled participants [73]. These findings suggest that butyrate-based interventions, including butyrate-producing bacterial supplementation, may ameliorate constipation-related symptoms through colonic mechanisms. A 2026 randomized placebo-controlled trial further evaluated the efficacy of microencapsulated sodium butyrate (1.5 g/day) in patients with type 2 diabetes complicated by IBS, confirming significant improvement in gastrointestinal symptoms. Through delayed-release technology, butyrate is ensured to reach the ileocecal region and colon directly, avoiding rapid metabolism of butyrate in the upper digestive tract [74]. Additionally, a randomized controlled trial of calcium butyrate in children with IBS showed that butyrate supplementation achieved marked clinical improvement in more than 70% of the enrolled children (51), indicating the therapeutic potential of butyrate preparations across different age groups [75].

### 4.3. Fecal Microbiota Transplantation and Precision Flora Reconstruction

FMT achieves efficient colonization of butyrate-producing bacteria and reconstruction of gut microbiota by transplanting complete flora from healthy individuals, demonstrating long-term efficacy in the treatment of refractory IBS. Johnsen et al. conducted a randomized, double-blind, placebo-controlled trial enrolling 83 moderate-to-severe IBS patients. Through colonoscopy, 50–80 g of mixed donor feces were transplanted into the patients’ colons. The FMT group showed significant symptom improvement during follow-up, with increased gut microbiota diversity [76]. El-Salhy’s team conducted follow-up studies on a cohort of 165 IBS patients at 2 and 3 years post-FMT, showing that fecal butyrate levels in the 30 g and 60 g groups remained significantly higher than baseline and control group levels at 2 and 3 years, and responders’ butyrate levels remained consistently higher than non-responders [77]. This finding indicates that FMT-induced butyrate-producing bacteria colonization and butyrate-level elevation possess long-term stability. Selection of high-quality donors is key to FMT success: donors should harbor a healthy gut microbiota and be enriched in butyrate-producing bacteria such as *Faecalibacterium prausnitzii* and *Eubacterium biforme*. In the future, screening donor feces with high abundances of butyrate-producing bacteria or constructing standardized butyrate-producing bacterial consortium preparations may further improve the precision and efficacy of FMT.

### 4.4. Lifestyle Intervention

Lifestyle influences the function of butyrate-producing bacteria indirectly by affecting gut microbiota structure and has synergistic effects with dietary intervention.

Exercise Intervention: Regular exercise is an important non-pharmacological means of regulating gut microbiota and butyrate-producing bacteria. Allen et al. conducted a randomized controlled trial in which 32 sedentary adults (18 lean, 14 obese) underwent 6 weeks of supervised endurance exercise training (60–75% heart rate reserve, three times per week) in a metabolic ward while maintaining a constant-calorie diet [78]. Results showed that fecal butyrate concentrations significantly increased in the lean group, with elevated abundance of butyrate-producing bacteria such as *Faecalibacterium*, whereas no significant changes were observed in the obese group, suggesting that host metabolic status influences the response of flora to exercise. Munukka et al. also reported that 6 weeks of moderate-intensity exercise increased intestinal *Akkermansia muciniphila* abundance and reduced the proportion of conditional pathogen *Proteobacteria* in sedentary overweight women [79]. Notably, the regulatory effects of exercise on flora are reversible: Allen et al. found that after 6 weeks of detraining, most exercise-induced flora and metabolic changes returned to baseline levels, with only fecal acetate remaining at higher levels in lean individuals, suggesting that long-term adherence to exercise is necessary to maintain benefits [78].

Psycho-Intestinal Regulation: Chronic psychological stress activates the hypothalamic–pituitary–adrenal (HPA) axis to release cortisol, alters gut microbiota composition, and inhibits butyrate-producing bacteria growth. Chen et al. conducted a cross-sectional study of 37 long-term deeply meditating Tibetan Buddhist monks and 19 neighboring non-meditating residents, showing that the meditation group had significantly higher abundance of intestinal *Prevotella*, *Bacteroides*, and *Faecalibacterium* than the control group, along with lower plasma total cholesterol and apolipoprotein B levels, suggesting that long-term meditation may indirectly promote butyrate synthesis by optimizing flora structure [80]. Dutcher et al. further reported in a randomized controlled trial that smartphone-based mindfulness meditation training reduced pro-inflammatory gene expression in stressed adults [81]. Although direct intervention studies quantifying “meditation improving butyrate synthesis efficiency” are currently lacking, existing evidence suggests that emotional regulation methods such as meditation and deep breathing can create a favorable intestinal microenvironment for butyrate-producing bacteria by enhancing vagal tone and reducing the negative impact of stress hormones on flora.

In summary, butyrate-producing bacteria-targeted interventions have formed a multi-dimensional system encompassing prebiotics, probiotics, FMT, and lifestyle regulation. Future large-sample, long-term randomized controlled trials are needed to clarify dose–effect relationships, individual response differences, and combined application strategies for different intervention modalities, to achieve precision microecological treatment of intestinal motility disorders.

## 5. Research Prospects and Challenges

### 5.1. Strain-Specific Effects and Synthetic Microbiomics

The strain-specific effects of butyrate-producing bacteria remain incompletely understood. The regulatory differences in different strains on intestinal motility and their synergistic mechanisms require further exploration. Vital et al. established a gene catalog containing 225 potential butyrate-producing bacteria by screening 3184 bacterial genomes, discovering that butyrate-producing capacity is inconsistent at the family level and cannot be accurately predicted relying solely on 16S rRNA sequencing [6]. Clark et al. further developed a model-guided synthetic human gut microbiome design method, quantifying the respective influences of microbial interactions on growth and butyrate production, identifying hydrogen sulfide, environmental pH, and resource competition as key molecular factors driving butyrate production [82]. This model can accurately predict community assembly and butyrate production at different species richness levels, providing a theoretical framework for constructing high-yield butyrate synthetic bacterial communities. Additionally, pan-genomic studies of *Anaerostipes hadrus* revealed functional diversity among butyrate-producing strains; its butyrate synthesis genes are as prevalent as 5-fluorouracil metabolism genes, suggesting that strain functionality is multifaceted. Future research should utilize metagenomic technologies to systematically screen highly active butyrate-producing strains, analyze their functional genes and ecological regulatory networks, and clarify the specific roles of different strains in intestinal motility regulation [83].

### 5.2. Gut–Brain Axis Mechanisms and FFAR2/FFAR3 Signaling Pathways

The specific conduction pathways of butyrate in the gut–brain axis and its interaction with the central nervous system require further in-depth investigation. Mishra et al. found that reduced butyrate-producing bacteria and decreased butyrate levels in the aging intestine subsequently inhibit the expression of free fatty acid receptors 2 and 3 (FFAR2/FFAR3), weaken mucin formation, increase intestinal permeability (“leaky gut”), and ultimately induce intestinal and cerebral inflammation and cognitive dysfunction [84]. This study reported that young mice with intestinal-specific knockout of *Ffar2* or *Ffar3* exhibited intestinal and cerebral abnormalities similar to aging mice, while butyrate supplementation could reverse these phenotypes by activating FFAR2/FFAR3 signaling. This suggests that FFAR2/FFAR3 agonists may be a potential strategy for alleviating aging-related gut–brain axis damage. Furthermore, butyrate may modulate gut–brain signaling through FFAR3 on intestinal neurons [85], although direct evidence for vagal afferent activation in this context remains limited. Future research should further clarify the specific circuits through which butyrate affects central intestinal motility regulatory nuclei via vagal afferent fibers, as well as its regulatory mechanisms on blood–brain barrier permeability and microglial activity.

### 5.3. Precision Microbiome Medicine and Personalized Intervention

Precision intervention strategies for different ages, sexes, and disease subtypes lack sufficient clinical evidence, and large-sample multi-center studies are urgently needed. A 2026 review systematically evaluated the diagnostic and therapeutic potential of microbiome analysis in precision medicine for gastrointestinal diseases, noting that although microbiome alterations show consistent associations with IBS, IBD, and other diseases, current evidence remains predominantly observational, and no microbiome test has received regulatory approval as an independent diagnostic tool [86]. At the precision treatment level, autoprobiotics—strains isolated from the patient’s own intestine, cultured in vitro, and then re-administered—have shown potential in pilot clinical trials for IBS, significantly improving dyspepsia symptoms and increasing the abundance of beneficial bacteria such as *Coprococcus* and *Blautia* [87]. In the future, a personalized diagnosis and treatment system based on gut microbiota detection should be established, combining 16S rRNA sequencing, metagenomics, and metabolomics to achieve closed-loop management of “flora typing—precision intervention.”

### 5.4. Engineered Butyrate-Producing Bacteria and Synthetic Biology

Synthetic biology provides new ideas for functional enhancement of butyrate-producing bacteria. Clark et al.’s data-driven model has proven that butyrate production in synthetic bacterial communities can be optimized through design–test–learn cycles [82]. Regarding engineered strain construction, researchers have reconstructed butyrate synthesis pathways in *Saccharomyces cerevisiae* through gene editing and regulated relevant metabolic modules to enhance butyrate production under anaerobic conditions, constructing engineered yeast strains capable of producing up to 1.8 g/L butyrate. This engineered yeast can regulate the gut microbiota of IBD patients in vitro, increase probiotic abundance, and demonstrate significant protective effects in TNBS-induced colitis mouse models. Notably, among this series of engineered yeasts, the strain with the second-highest butyrate synthesis capacity showed the most significant regulatory effects on the gut microbiota of IBD patients and colitis mouse models [88]. Additionally, intervention strategies based on engineered probiotics are also being explored. For example, synthetic bacterial systems constructed using EcN and other chassis strains can achieve targeted release of therapeutic payloads by sensing intestinal microenvironment signals (such as pH, hypoxia, or specific metabolites), providing new ideas for precision regulation of gut microbiota [89]. Future research should focus on developing more targeted intervention methods, such as butyrate receptor agonists, strain-specific prebiotics, and programmable butyrate-producing engineered bacteria.

### 5.5. Age-Related Changes and Life-Cycle Interventions

Age is a key variable affecting the function of butyrate-producing bacteria. Chilton et al. reported in the 3 × Tg-AD mouse model that age-related temporal decline in butyrate-producing bacteria plays a key pathogenic role in the onset and progression of neuropathology and memory deficits [90]. A 2023 large-scale metagenomic analysis further revealed [91] that butyrate production pathway abundance is nearly zero during infancy, then steadily increases with age, whereas propionate pathways show the opposite trend; delivery mode also significantly affects propionate pathway abundance during infancy (higher in vaginal delivery than cesarean section). These findings suggest that butyrate-producing bacteria intervention strategies should differ across life-cycle stages: infancy should focus on flora colonization and cultivation, adulthood on maintaining homeostasis, and old age on compensating for butyrate-producing bacteria loss. Mishra et al.’s research further indicates that butyrate supplementation can restore aging intestinal barrier function and improve brain health, providing experimental evidence for the intervention of intestinal motility disorders in the elderly [84].

### 5.6. Butyrate Receptor-Targeted Drug Development

FFAR2 and FFAR3, as the primary receptors for butyrate, are important targets for drug development in intestinal motility disorders. Mishra et al.’s research clearly reported that FFAR2/FFAR3 agonists can alleviate intestinal and brain function damage induced by aging gut microbiota [84]. Although the application of FFAR2 agonists in human studies is currently limited by differences in human–mouse receptor signal transduction, with advances in receptor structural biology and medicinal chemistry, developing small-molecule agonists with high selectivity and tissue specificity for FFAR2/FFAR3, or designing prodrugs that can target and activate intestinal local receptors, will become important directions for precision treatment of intestinal motility disorders [92].

In summary, research on butyrate-producing bacteria is progressing from descriptive associations toward mechanistic analysis and precision intervention. Future efforts should integrate multi-omics technologies, synthetic biology, and clinical epidemiology to establish dynamic intervention models based on individual flora characteristics on the basis of resolving strain–host–environment interaction networks, ultimately achieving precision microecological treatment of intestinal motility disorders.

## 6. Limitations

This review has several limitations. First, much of the mechanistic evidence derives from rodent models, and species differences in FFAR2/FFAR3 pharmacology limit direct translation to humans [93]. Second, fecal SCFA concentrations are commonly used as a surrogate for colonic butyrate production, yet they reflect the net of synthesis, microbial consumption, epithelial absorption, and transit time rather than true local production [94]. Third, complex interventions such as FMT, resistant starch, and dietary fiber make it difficult to attribute clinical effects specifically to butyrate rather than to concurrent changes in other metabolites or taxa [95]. Fourth, the included clinical trials are heterogeneous in design, population, intervention dose, duration, and endpoints, and many are small single-center studies; consequently, risk-of-bias and publication bias should be considered when interpreting their findings [96]. Finally, the taxonomy of butyrate-producing bacteria is evolving, and some family-level assignments used in older literature may no longer reflect current classifications [97].

## 7. Conclusions

Butyrate-producing bacteria regulate intestinal motility through multiple pathways, including enteroendocrine hormone release, enteric nervous system modulation, and ICC/smooth muscle activity. Clinical evidence supports targeted interventions such as prebiotics, live biotherapeutics, and FMT, but heterogeneity among trials and the imperfect nature of fecal SCFA as a surrogate for colonic butyrate production limit causal inference. Future studies should integrate multi-omics, standardized taxonomy, and well-designed clinical trials to advance precision microbiome-based therapies for intestinal motility disorders.

## Data Availability

No new data were created or analyzed in this study. Data sharing is not applicable to this article.

## Abbreviations

ACh	Acetylcholine
AAV	Adeno-associated viral vector
AbfD	4-hydroxybutyryl-CoA dehydratase
AbfH	4-hydroxybutyrate dehydrogenase
atoB	acetyl-CoA acetyltransferase gene (thiolase A)
Bcd	butyryl-CoA dehydrogenase
BDNF	brain-derived neurotrophic factor
Buk	butyrate kinase
But	butyryl-CoA:acetate CoA-transferase
c-Fos	cellular Fos proto-oncogene
ChAT	choline acetyltransferase
CNO	clozapine N-oxide
CUMS	chronic unpredictable mild stress
DRG	dorsal root ganglion
EC	enterochromaffin cell
EEC	enteroendocrine cell
EGC	enteric glial cell
ENS	enteric nervous system
FFAR2	free fatty acid receptor 2
FFAR3	free fatty acid receptor 3
FMT	fecal microbiota transplantation
GABA	γ-aminobutyric acid
Gcg	proglucagon
Gct	glutaconate CoA-transferase
GF	germ-free
GFAP	glial fibrillary acidic protein
GDNF	glial cell line-derived neurotrophic factor
Gdh	glutamate dehydrogenase
Hgd	2-hydroxyglutarate dehydrogenase
Hbd / BHBD	3-hydroxybutyryl-CoA dehydrogenase
HDAC	histone deacetylase
HPA	hypothalamic–pituitary–adrenal axis
IBD	inflammatory bowel disease
IBS	irritable bowel syndrome
IBS-C	constipation-predominant irritable bowel syndrome
IBS-D	diarrhea-predominant irritable bowel syndrome
ICCs	interstitial cells of Cajal
IL-1β	interleukin-1β
IL-6	interleukin-6
JNK	c-Jun N-terminal kinase
KAT	class I thiolase
Kce	3-keto-5-aminohexanoate cleavage enzyme
KamA	lysine-2,3-aminomutase
Kgr	α-ketoglutarate reductase
MCT2	monocarboxylate transporter 2
MLC	myosin light chain
NF-κB	nuclear factor-κB
NO	nitric oxide
NTS	nucleus tractus solitarius
phaA	acetoacetyl-CoA thiolase gene (thiolase B)
PVT	paraventricular thalamic nucleus
Ptb	phosphotransbutyrylase
PYY	peptide YY
SCFA	short-chain fatty acid
SAS	self-rating anxiety scale
SDS	self-rating depression scale
TPH1	tryptophan hydroxylase 1
TLR2	Toll-like receptor 2
TNF-α	tumor necrosis factor-α
VIP	vasoactive intestinal peptide
5-HT	5-hydroxytryptamine
5-HTR	5-hydroxytryptamine receptor
